# A liquid biopsy approach detects HCC and identifies GJA4 as a potential biomarker for HBV-HCC via plasma cfDNA methylome profiling

**DOI:** 10.1186/s13148-025-01909-w

**Published:** 2025-06-11

**Authors:** Jialing Sun, Xinfeng Sun, Weihuang He, Bingding Huang, Wenxing Feng, Zhiyi Han, Ruyun Ruan, Yuanke Pan, Jinxin Zhu, Jing Li, Xin Zhong, Mengqing Ma, Rui Hu, Minling Lv, Qi Huang, Wei Zhang, Mingji Feng, Jinyu Yi, Pin Cui, Xiaozhou Zhou

**Affiliations:** 1 Department of Liver Disease, the Fourth Clinical Medical School, Guangzhou University of Chinese Medicine, Shenzhen, 518033 China; 2 Department of Liver Disease, Shenzhen Traditional Chinese Medicine Hospital, Shenzhen, 518033 China; 3 Shenzhen Rapha Biotechnology Incorporate, Shenzhen, 518118 China; 4 College of Big Data and Internet, Shenzhen Technology University, Shenzhen, 518118 China

**Keywords:** HBV-HCC carcinogenesis, cfDNA, Methylation profile, Early detection, DMR

## Abstract

**Background:**

Early detection of hepatocellular carcinoma (HCC) can greatly improve the survival rate of patients. Plasma cfDNA methylation has been shown to have the potential to be a non-invasive method for diagnosing HCC. However, the identified HCC plasma cfDNA methylation sites were less sensitive to early HCC diagnosis. Therefore, we aimed to develop a highly sensitive marker panel based on cell-free DNA (cfDNA) methylation for the detection of HCC.

**Methods:**

The study included 374 participants, including 102 healthy individuals, 51 HBV patients, 50 cirrhosis patients, and 171 HCC patients (56 at stage 0 or A according to BCLC staging). Two cfDNA methylation sequencing assays (whole genome bisulfite sequencing (WGBS) and targeted bisulfite sequencing (TBS)) were used along with machine learning modeling to detect HBV-related HCC based on differentially methylated regions (DMR) among the four participant groups.

**Results:**

TBS analysis achieved an overall sensitivity of 96.67% at a specificity of 93.7% than alpha-fetoprotein (AFP) of 18%-60%, to discriminate all stages of HCC patients from healthy people, and sensitivity of 90.0% at a specificity of 93.75% to discriminate early-stage HCC patients from healthy people. A number of significant DMRs between HCC and non-cancer groups were identified, providing candidate biomarkers for HCC detection. Among these DMRs, one that locates in the promoter region of *GJA4*, was found to be consistently present in the whole process of HBV-related HCC carcinogenesis. Using data from TCGA, comparison of expression profile of *GJA4* between 160 healthy people and 369 HCC patients further supported this scenario.

Conclusions: This study provides biomarkers for detecting, staging and early detection of HCC using plasma cfDNA methylome profiling. Additionally, the dynamic alteration of GJA4 promoter methylation may serve as a molecular clue for studying HBV-related HCC carcinogenesis and prognosis.

**Supplementary Information:**

The online version contains supplementary material available at 10.1186/s13148-025-01909-w.

## Introduction

The World Health Organization reports that primary liver cancer (PLC) is the sixth most common form of malignant tumors, with the third highest mortality rate [1]. The majority (85%) of primary liver cancer (PLC) cases are constituted by hepatocellular carcinoma (HCC), with a relatively low five-year survival rate for patients ranging from 10 to 19% [2]. Hepatitis B virus (HBV) infection is a key risk factor for HCC. Chronic HBV infection leads to liver inflammation and fibrosis, which progresses to cirrhosis and eventually HCC. HBV-related HCC accounts for more than 80% of the total number of HCC patients globally. The pathogenesis of HBV-related HCC involves epigenetic mechanisms, especially DNA methylation [3]. Upon diagnosis, the majority of HCC patients are already categorized as stage C or D based on the Barcelona Clinic Liver Cancer (BCLC) staging system, rendering radical curative approaches such as surgical resection unviable. The five-year survival rate for HCC at BCLC stage A can reach up to 70%, in contrast. Liver ultrasound and serum alpha-fetoprotein (AFP) testing every 6 months are the primary screening strategies for HCC in high-risk populations [4]. The accuracy of AFP, however, is relatively low, with a specificity ranging from 85 to 90% and a sensitivity ranging from 18 to 60%, making it particularly ineffective for the early detection of liver neoplasms [5]. Hence, it is imperative to develop precise and cost-effective methods for early-stage HCC detection, which poses significant challenges.

Research has found that liquid biopsy based on cell-free DNA (cfDNA) has become a promising non-invasive method for the diagnosis, prognosis and monitoring of cancer. DNA methylation alterations occur in the early stage of tumorigenesis, even before somatic mutations appear, which also provides an opportunity to identify early-stage cancer before clinical symptoms appear. Many studies have shown that the detection of plasma cfDNA methylation has strong application potential for the diagnosis of HCC. However, the sensitivity of the currently discovered cfDNA methylation markers for the diagnosis of early HCC is still relatively low (less than 80%) [6, 7], and there is a need for further improvement. Whole genome bisulfite sequencing (WGBS) can precisely detect the methylation level of all individual cytosine bases (C bases) across the entire genome and is the gold standard for DNA methylation research [8]. Based on this, this study aims to successfully achieve precise and cost-effective detection and stratification of HCC through the combination of WGBS and targeted bisulfite sequencing (TBS) technology with machine learning-based classifiers, thereby identifying multiple biomarkers for the early diagnosis of HCC and gaining a deeper understanding of the pathogenesis of HBV-related HCC.

## Materials and methods

### Study Design and participants enrollment

**Fig. 1:**
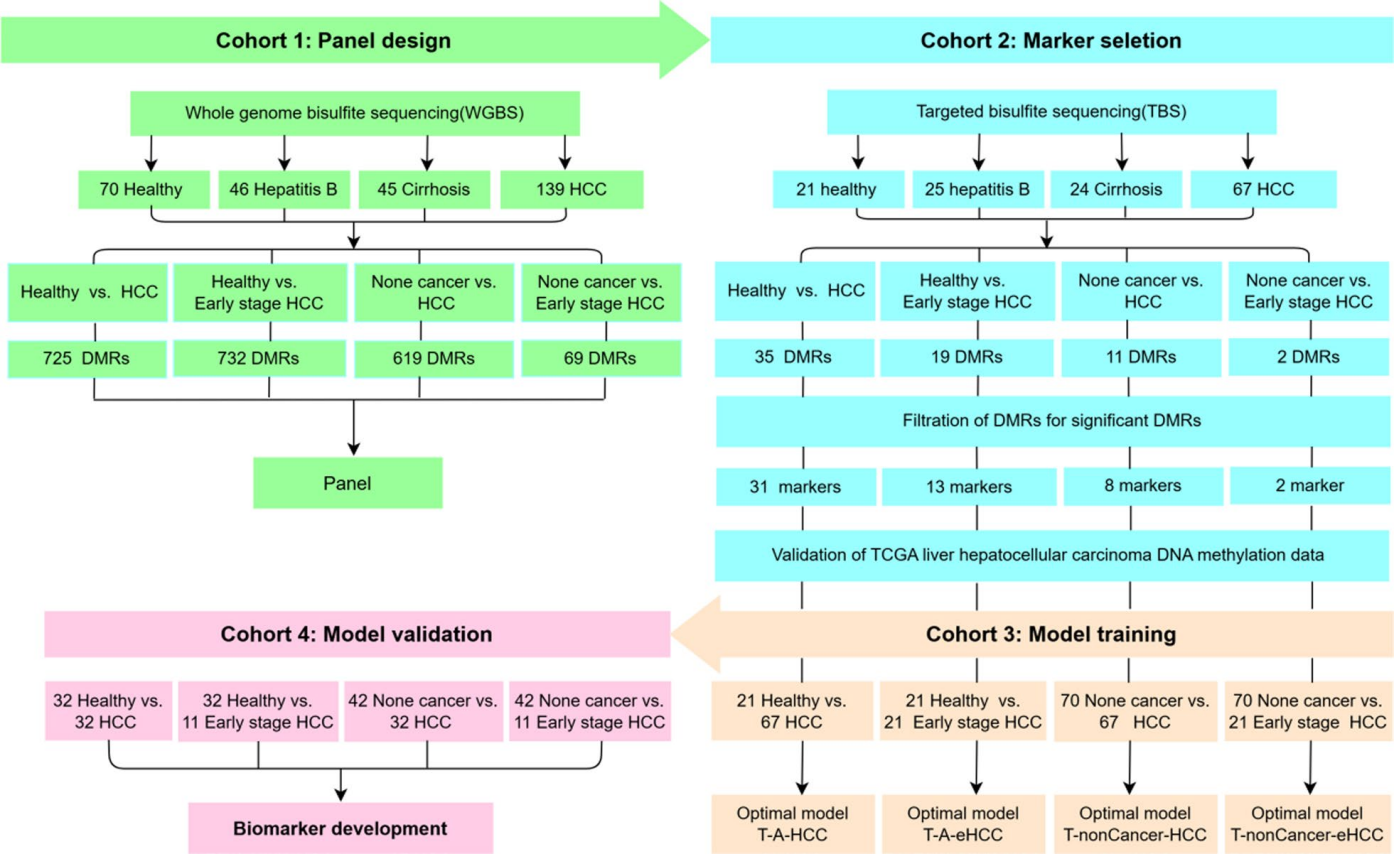
The general workflow of this study

The enrollment of all patients took place upon diagnosis at Shenzhen Traditional Chinese Medicine Hospital. The study protocols were approved by the ethics committee of Shenzhen Traditional Chinese Medicine Hospital (Approval No.: [2018]58) and conducted in accordance with internationally recognized standards of good clinical practice. Before taking part, all participants were given detailed study information and provided their consent after being fully informed. The designated pipeline (Fig. 1) was adhered to for conducting experimental procedures and analyzing the data. This study was conducted with a recruitment of 374 individuals, divided into four cohorts (Fig. 1). The data regarding the patient is displayed within Table S1. All patients diagnosed with chronic HBV infections were clinically confirmed to have cirrhosis and HCC. As an initial investigation, Cohort 1 comprised of 70 individuals in good health, 46 patients with chronic hepatitis B, 45 patients diagnosed with cirrhosis, and a total of 139 patients suffering from HCC. In the first cohort, we conducted WGBS on these samples to identify distinct regions with differentially methylated regions (DMRs) between non-cancer and HCC groups. Subsequently, a panel was devised specifically targeting genomic areas associated with these DMRs. To conduct a comprehensive evaluation of the diagnostic effectiveness of these DMRs, we enrolled Cohort 2 consisting of 21 individuals with no health issues, 25 patients diagnosed with chronic hepatitis B, 24 patients diagnosed with cirrhosis, and 67 patients diagnosed with HCC (including 21 cases in the early stage). All samples from Cohort 2 were subjected to TBS using the panel derived from Cohort 1, leading to the discovery of several DMRs by comparing various groups. DMRs that exhibited a *p* value < 0.05 and an absolute discrepancy ≥ 0.1 were considered significant, and these markers were utilized for constructing the model in Cohort 3 and validating it in Cohort 4. Cohort 3 employed identical samples to Cohort 2, whereas Cohort 4 incorporated an additional group of participants consisting of 32 individuals in good health, 4 patients with chronic hepatitis B, 6 patients with cirrhosis, and a total of 32 patients diagnosed with HCC (including 11 patients at the early stage). Patients with HCC in the early stages were classified as stage 0 or A based on the BCLC staging system.

### CfDNA extraction from plasma samples

The plasma was acquired by subjecting the whole blood to centrifugation at a speed of 1,600 g for a duration of 10 min. The supernatant was subsequently transferred to a new tube and subjected to centrifugation at 10,000 g for 15 min in order to eliminate cellular debris from the plasma. CfDNA was isolated from 3 mL of plasma per participant utilizing the HiPure Circulating DNA Midi Spin Kit S (Magen Biotech Inc., Guangzhou, China). The final elution volume was 50 μL after purification. The quality control (QC) process for the libraries involved utilizing Qsep100 (Bio-optic, Inc., Taiwan, China) to evaluate the distribution of fragment sizes and employing Qubit 4.0 (Thermo Fisher Inc., Waltham, MA, USA) to determine the concentration.

### WGBS library construction and sequencing

The WGBS analysis for Cohort 1 involved utilizing 10–40 ng of cfDNA input per participant. Bisulfite conversion is a necessary and standard procedure prior to conducting WGBS, during which cytosine that are not methylated undergo chemical modification to become uracil. These modified bases are then identified as thymine during the sequencing phase. To ensure optimal conversion efficiency, a critical QC parameter in WGBS, we employed the EpiArt DNA Methylation Bisulfite Kit (Vazyme Biotech Inc., Nanjing, China) for bisulfite conversion. This kit is renowned for achieving a minimum conversion rate of 99%. The WGBS libraries were prepared for cfDNA samples applying the RainbowMerry cfDNA Methylseq Library Preparation Kit (Rapha Biotech. Inc., Shenzhen, China). The libraries were subjected to QC analysis employing Qsep100 (Bio-optic. Inc., Taiwan, China) and Qubit 4.0 (Thermo Fisher. Inc., MA, USA). Pools from each set of eight libraries were individually subjected to sequencing on a distinct lane of an MGI-2000 sequencer, utilizing DNBSEQ technology and PE100 sequencing mode.

### WGBS data processing

The raw data obtained from WGBS were subjected to QC protocol, which included filtration using fastp [9]. After base recognition, cutadapt (v 1.8.3) was employed to trim all paired terminal fastq files to remove adapter sequences and low-quality bases (the quality of bases was lower than Q20, and the minimum length of reads was 36). The rate of bisulfite conversion was assessed by quantifying the percentage of cytosines that underwent transformation into thymines within the Lambda DNA. It is important to note that all cytosines present in this particular sample were found to be devoid of methylation. The eligible reads were then mapped to the human reference genome (GRCh38.p14) utilizing the BWA sequence aligner [10], after eliminating PCR duplicates with SAMtools [11] and sambamba [12]. Subsequently, MethyDackel was utilized to assess the methylation levels of CpG sites comprehensively, whereas Metilene was employed for the detection and characterization of differentially methylated regions (DMRs) [13]. Significant DMRs were utilized to generate genome-wide methylation profiles, which were visually represented using NG-Circos.

### DMRs based TBS deep sequencing

To conduct a comprehensive analysis of methylation discrepancies between individuals without cancer and those with HCC, we employed targeted bisulfite sequencing (TBS) to deeply sequence a set of genomic regions associated with DMRs. To develop this panel, we processed 300 WGBS samples from Cohort 1 using the aforementioned method. Subsequently, DMR analysis identified a total of 725 DMRs distinguishing healthy individuals from HCC patients, 619 DMRs differentiating the non-cancer group from HCC patients, 732 DMRs discriminating healthy individuals from early-stage HCC patients, and finally, 69 DMRs separating the non-cancer group from early-stage HCC patients. These DMRs served as the basis for developing our tailored panel, which encompasses 461 kb of the human genome and incorporates a total of 827 CpG islands. The initial step of the TBS assay involved constructing pre-capture libraries using the identical procedure as described above for WGBS library construction, encompassing a sample indexing strategy and incorporation of Lambda DNA as an internal control. For the TBS data, the initial processing and detection of noteworthy DMRs were conducted using a similar approach as employed for the WGBS data.

### Selection of DMRs as candidate markers for HCC detection

In Cohort 2, we observed a total of 31 DMRs that showed significant differences between healthy individuals and HCC patients. Additionally, there were 8 DMRs that distinguished non-cancer group from HCC patients. Furthermore, we identified 13 DMRs that exhibited variations between healthy individuals and early-stage HCC patients, while only 2 DMRs were found to be differentiating the non-cancer group from early-stage HCC patients. These noteworthy DMRs were regarded as potential indicators for the identification of HCC. What’s more, we conducted a comparative analysis of the methylation sequencing data from 747 healthy individuals and 430 patients diagnosed with HCC in the TCGA database. Our findings revealed variations in methylation levels within genomic regions associated with these notable DMRs. These important DMRs were annotated using Annovar software, and then gene enrichment analysis was performed using R-package clusterProfiler [14] to identify relevant genes for biological interpretation.

### Prediction models for HCC detection

The feature markers derived from the TBS data (in Cohort 2) that exhibited significant DMRs were utilized to develop prediction models in Cohort 3, employing identical samples as those used in Cohort 2. Four different prediction models were established to serve distinct purposes. These include the T-H-HCC model, which aims to differentiate between healthy individuals and HCC patients; the T-nonCancer-HCC model, designed to distinguish all non-cancer participants from HCC patients; the T-H-eHCC model, focused on discriminating healthy individuals from early-stage HCC patients; and finally, the T-nonCancer-eHCC model, aimed at distinguishing all non-cancer participants from those with early-stage HCC. In each of these models, the TBS data for both parties being compared were divided into a training set and a test set using a random allocation ratio of 7:3. These models were constructed utilizing the XGBoost machine learning algorithm [15]. The training parameters for the models were configured as follows: 1000 estimators, a maximum depth of 2, a learning rate of 0.05, and five-fold cross-validation (n_estimators = 1000, max_depth = 2, learning_rate = 0.05, nfold = 5). The models undergo multiple iterations to achieve optimal performance. Furthermore, Cohort 4 was enlisted to simulate a real-life scenario in the identification of HCC. The feature markers identified from Cohort 2's significant DMRs and the optimal models trained in Cohort 3 were utilized to train four prediction models. These models were then validated using all TBS samples from Cohort 4 as a test set, while the training set consisted of all TBS samples from Cohort 3.

Numpy (v 1.18.5) was utilized to compute the sensitivity(TP/[TP + FN]), specificity(TN/[TN + FP]), and accuracy([TP + TN]/[TP + FP + TN + FN]) based on the values of true-positive(TP), true-negative(TN), false-positive(FP), and false-negative(FN). The cut-off value for generating the receiver operating characteristic (ROC) curve was determined using the sklearn package in Python, resulting in obtaining the area under the ROC curve (AUC).

### Biomarkers for HBV-related HCC carcinogenesis

To explore the molecular mechanisms involved in the carcinogenesis of HBV-related HCC, we amalgamated samples from Cohort 3 and Cohort 4 and conducted a differential methylation analysis on TBS data obtained from 53 healthy individuals, 29 patients with HBV infection, 30 cirrhotic patients, and 99 HCC patients. We discovered a DMR that plays a role in the progression of HBV, serving as a potential biomarker for HCC associated with HBV carcinogenesis. To assess the validity of this potential biomarker, we analyzed the expression patterns of the gene associated with this DMR in a cohort of 369 HCC patients and 160 healthy controls from the TCGA database.

## Results

### Genome-wide methylation profiles of different groups

**Fig. 2:**
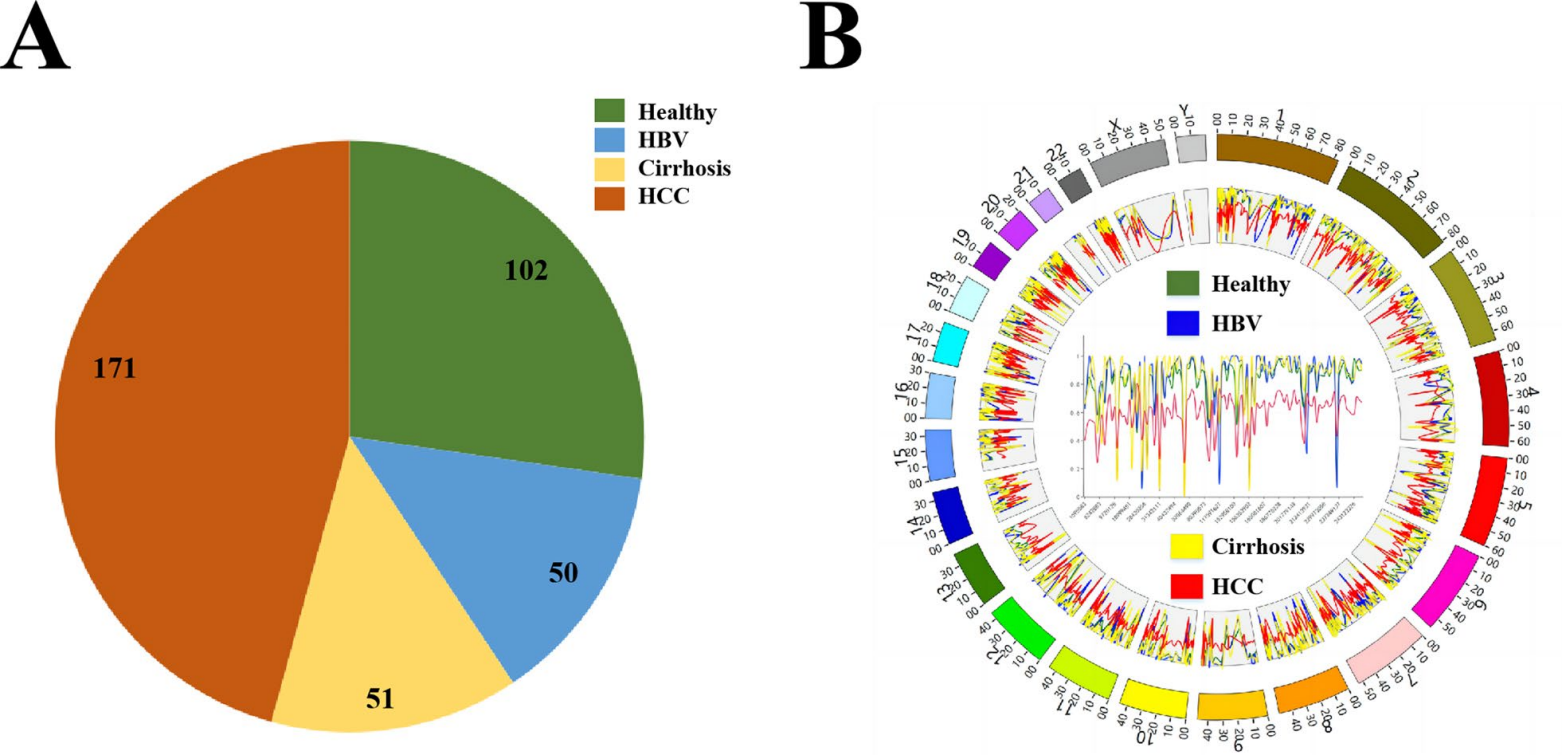
A circos plot of cfDNA methylation levels in DMRs between different groups. **A** The distribution of participants across different groups. **B** The methylation levels of healthy group(green line), hepatitis B group (blue line), cirrhosis group (yellow line), and HCC group (red line), and we also plotted the methylation levels of the four groups across Chromosome 1 inside this circos plot to show higher-resolution methylation profiles

This study involved a total of 374 participants, with the distribution of participants across different groups was illustrated in Fig. 2A. The QC analysis of the WGBS data in Cohort1 revealed cytosine conversion rates from 98.2% to 100%, with an average of 98.97%, which is within the normal range, ensuring the reliability and accuracy of bisulfite sequencing. DMRs were identified through comparison of different groups within the WGBS dataset. Figure 2B demonstrates significant variations exist between the methylation levels of four groups of participants in DMR-related genomic regions. Notably, HCC patients exhibited genome-wide hypomethylation compared to the other groups, except for sporadic peaks distributed throughout the genome.

### HCC staging based on significant DMRs

**Fig. 3:**
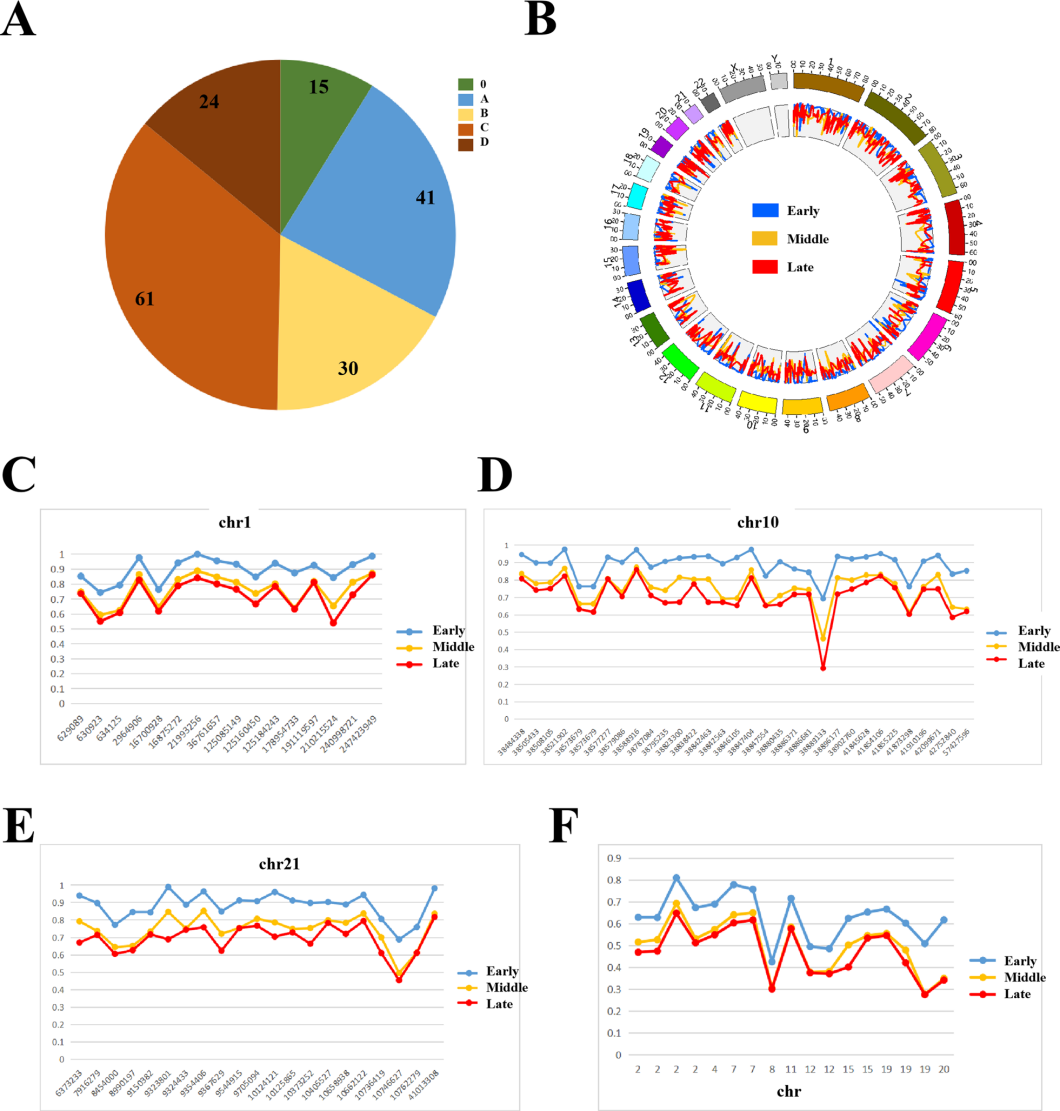
Comparison of the methylation levels of HCC patients at different stages. The blue line represents the early category, the yellow line represents the middle category, and the red line represents the late category. **A** The distribution of HCC patients in different stages. **B** Genome-wide methylation levels of HCC patients at different stages in the WGBS data. **C**-**E** Methylation levels of HCC patients at different stages in the WGBS data on specific chromosomes. DMRs represented the level of methylation difference at each stage. **F** Genome-wide methylation levels of HCC patients at different stages in the TBS data

Staging of HCC is essential for accurate diagnosis and effective treatment, traditionally relying on clinical observation and adhering to the BCLC staging system [16]. The distribution of HCC patients in different staging groups in this study was illustrated in Fig. 3A. To provide molecular evidence for staging, we compared the methylation levels between HCC patients at different stages. For clarity, we simplified the staging into three broad categories: early (stages 0 and A according to BCLC staging), middle (stage B according to BCLC staging), and late (stages C and D according to BCLC staging). Furtherly, in order to ensure statistical significance in our comparison, only significant DMRs were considered. Based on WGBS data, we conducted a genome-wide comparison of differentially methylated regions (DMRs) among HCC patients at various stages (Fig. 3B), as well as in specific chromosomal regions (Fig. 3C-3E). Similarly, based on TBS data, we performed a comprehensive analysis of DMRs between HCC patients at different stages (Fig. 3F). Our findings revealed distinct methylation level profiles across the three staging categories, with a global increase in hypomethylated regions observed in later stages.

### Prediction models for HCC detection based on TBS data

Based on the panel designed from DMRs identified by WGBS in Cohort 1, we performed TBS in Cohort 2. The target depth of TBS ranged from 403.69 to 8386.72 × , with an average sequencing depth of 2390 × , enabling an in-depth analysis of methylation levels. DMRs showing significant differences were selected as feature markers to construct prediction models for HCC detection based on TBS data in Cohort 3 (Table S2).

**Fig. 4:**
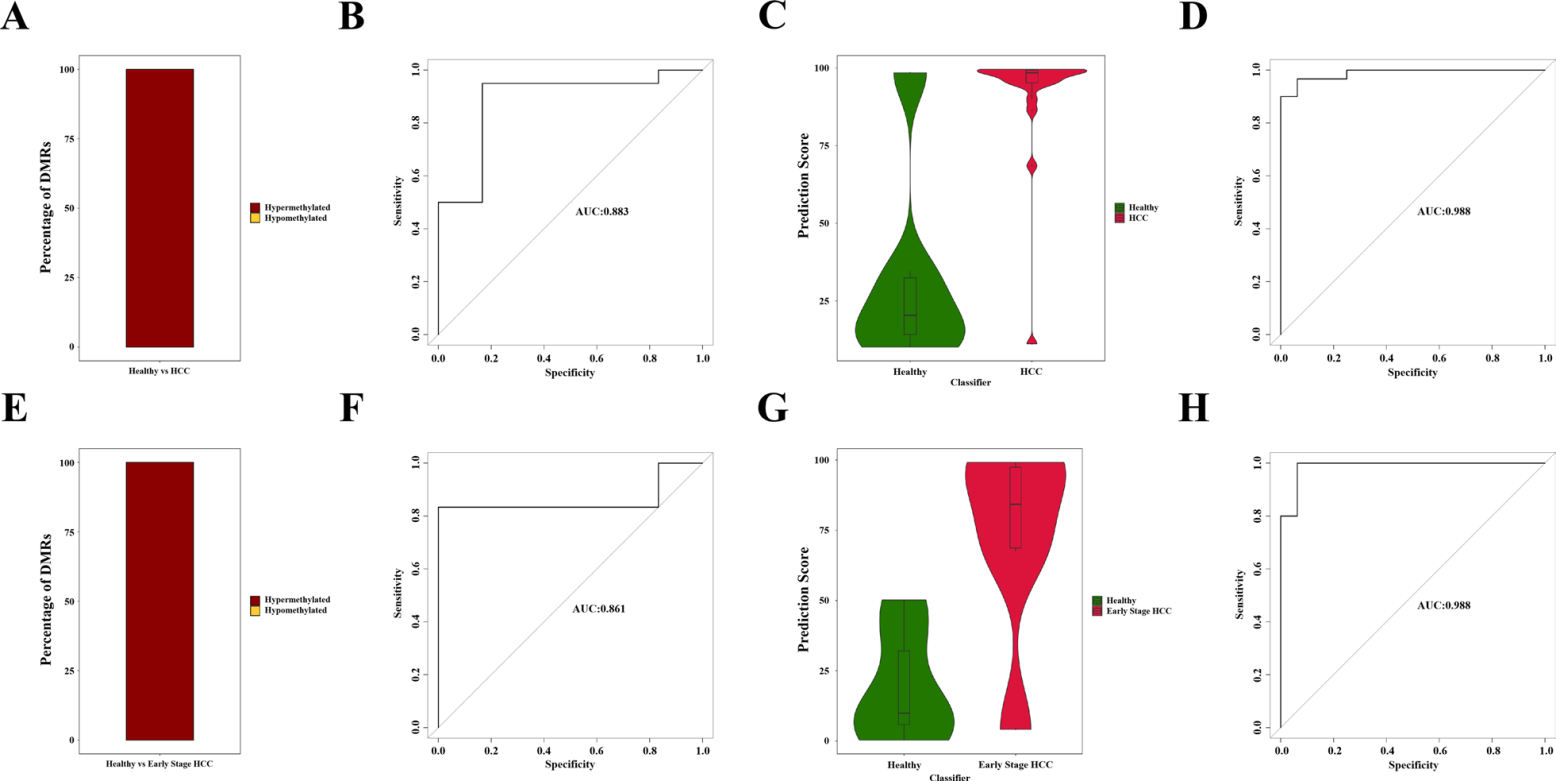
The T-A-HCC and T-A-eHCC prediction model developed using TBS data. **A** Decomposition of hyper- or hypo-methylation of significant DMRs between healthy and HCC groups; **B** Prediction scores for this model T-A-HCC; **C** ROC curve for building up of this model T-A-HCC; **D** ROC curve for validation of this model T-A-HCC; **E** Decomposition of hyper- or hypo-methylation of significant DMRs between healthy and early-stage HCC groups; **F** Prediction scores for this model T-A-eHCC; **G** ROC curve for building up of this model T-A-eHCC; **H** ROC curve for validation of this model T-A-eHCC

In Cohort 2, a total of 31 significant DMRs were obtained from the Healthy vs. HCC comparison, all exhibiting hypermethylation patterns (Fig. 4A). Similarly, in the Healthy vs. Early-stage HCC comparison, a total of 13 significant DMRs were observed, all displaying hypermethylation patterns (Fig. 4E). In Cohort 3, the T-A-HCC model achieved a sensitivity of 95.00%, a specificity of 83.33%, and an AUC of 0.883(Fig. 4B). The corresponding prediction scores were depicted in Fig. 4C. And the T-A-eHCC model achieved a sensitivity of 83.33%, a specificity of 83.33%, and an AUC of 0.861 (Fig. 4F), with the prediction scores shown in Fig. 4G.

Subsequently, utilizing the same significant DMRs as feature markers, we validated the T-A-HCC model by employing all healthy individuals and HCC patients from Cohort 3 as the training set and those from Cohort 4 as the test set, resulting in a high AUC of 0.988 (Fig. 4D). Furthermore, we validated the T-A-eHCC model using all healthy individuals and early-stage HCC patients from Cohort 3 as the training set and those from Cohort 4 as the test set, achieving a comparable AUC of 0.988 (Fig. 4H). The supplementary materials provide detailed analysis results for None cancer vs. HCC and None cancer vs. Early-stage HCC (Fig. S1).

### HCC specific methylation markers were identified based on significant DMRs

**Fig. 5:**
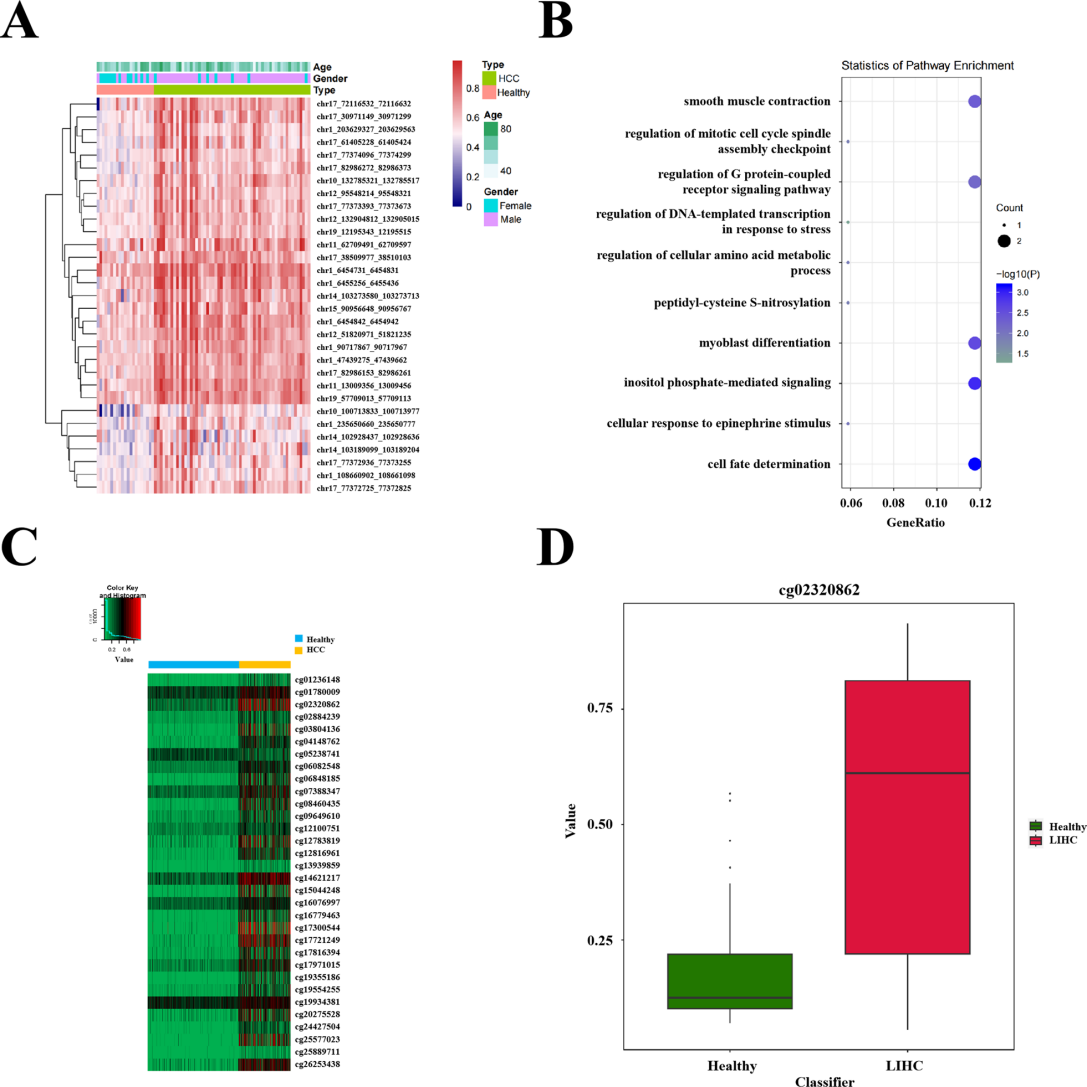
Biological interpretation of candidate biomarkers to discriminate HCC patients from healthy individuals. **A** Heatmap for unsupervised clustering of differential methylation levels of the 31 candidate biomarkers(significant DMRs) between healthy individuals and HCC patients identified from TBS data. The abscissa represents each of the samples, while the ordinate represents each of the 31 candidate biomarkers. **B** Enrichment analysis of gene ontology for candidate biomarkers. The abscissa represents the ratio of number of genes annotated against the total number of genes in each of the pathways. And the ordinates on the left are the names of GO pathways while ordinate on the right comprises -log10(P) and Count. **C** Heatmap for unsupervised clustering of differential methylation level between 747 healthy individuals and 430 HCC patients at the CpG sites which were located in regions of the 31 significant DMRs identified above. The abscissa represents each of the samples with light blue part for healthy individuals and pink part for HCC patients, while the ordinate represents the CpG sites. **D** Boxplot for the methylation values of 747 healthy individuals and 430 HCC patients at CpG site cg02320862. The abscissa represents each of the participants’ groups with green for healthy individuals and red for HCC patients, while the ordinates represents the methylation values of the two groups of participants at this CpG site

Based on differential methylation analysis of TBS data, a number of significant DMRs were identified. And these are no significant differences regarding age and gender among these DMRs (Fig. 5A). Which were proposed as candidate biomarkers for HCC detection. Using Annovar software to annotate 24 genes from the 31 significant DMRs found in Healthy vs. HCC comparison (Table S3), enrichment analysis via Gene Ontology (GO) revealed that these genes were involved in carcinogenic signal pathways related to transcription regulation, cell fate decision and regulation of oncogenes or tumor suppressor genes (Fig. 5B). Some of these genes were also found to be transcription factors, such as TBX2, PAX2, ZNF605, BARHL2 and ZNF154. Notably, TBX2, PAX2, and ZNF154 have been reported to play a role in the carcinogensis of HCC [16–18], providing biological plausibility for the biomarkers discovered in our study. Furthermore, we conducted a differential methylation analysis on the methylation profiles of 430 HCC patients and 747 healthy individuals from TCGA database (Fig. 5C-D). By generating a heatmap (Fig. 5C), we observed significant difference in methylation levels between these two groups at CpG sites within the genomic regions covered by the previously identified 31 significant DMRs. This provided further evidence that these 31 DMRs exhibited consistent hypermethylation in HCC patients while displaying dramatically hypomethylation in healthy population, suggesting their potential as biomarkers to discriminate HCC patients from healthy individuals.

**Fig. 6:**
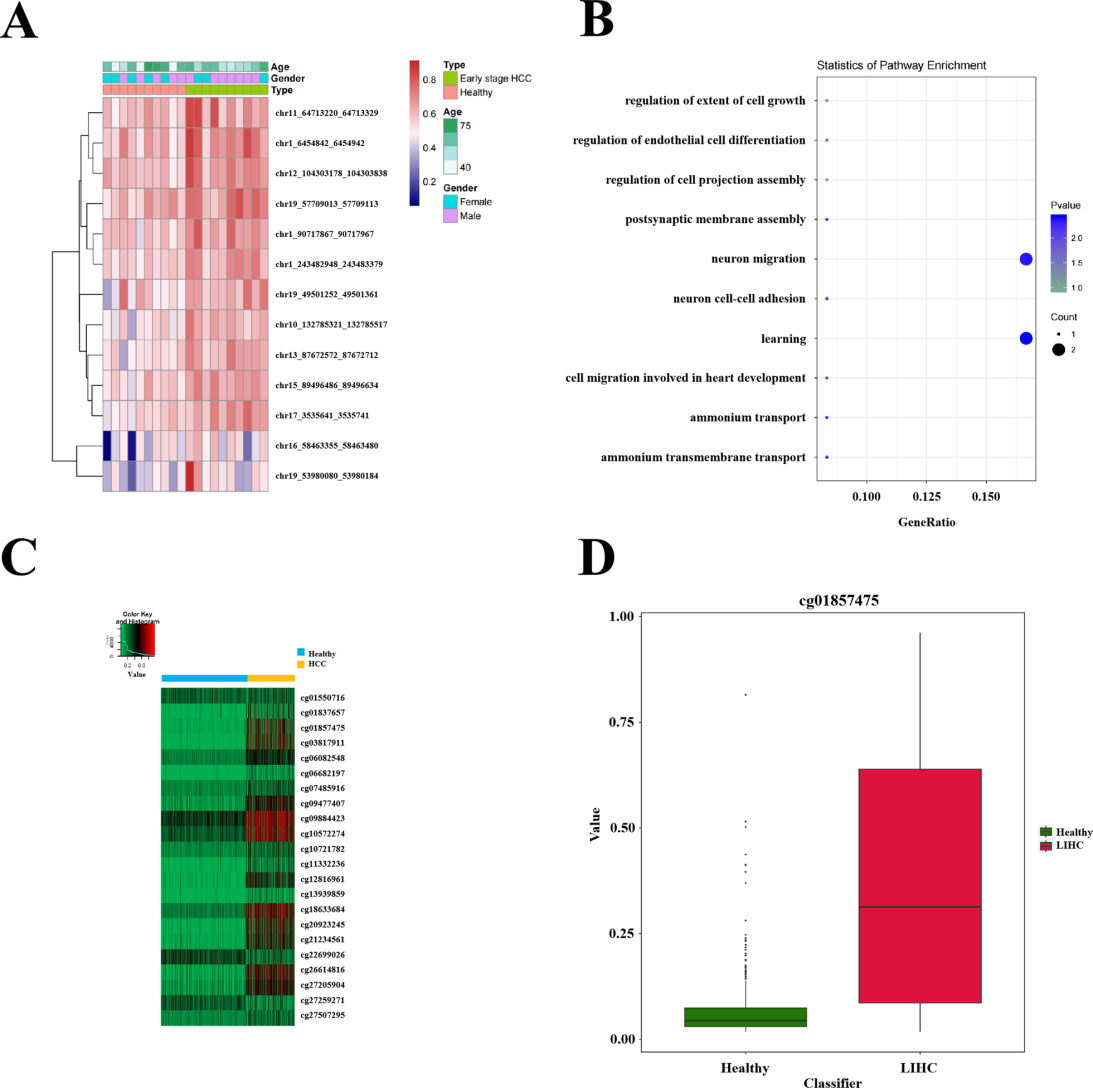
Biological interpretation of candidate biomarkers to discriminate early stage HCC patients from healthy people. **A** Heatmap for unsupervised clustering of differential methylation levels of the 13 candidate biomarkers(significant DMRs) between healthy individuals and early-stage HCC patients identified from TBS data. **B** Enrichment analysis of gene ontology for candidate biomarkers. **C** Heatmap for unsupervised clustering of differential methylation level between 747 healthy people and 430 HCC patients at the CpG sites which were located in regions of the 13 significant DMRs identified above. **D** Boxplot for the methylation values of 747 healthy people and 430 HCC patients at CpG site cg01857475

The 13 significant DMRs between healthy individuals and early-stage HCC patients correspond to 13 genes(Table S4). Enrichment analysis using GO revealed that these genes were involved in carcinogenic signal pathways (Fig. 6A-B). Notably, EID3, MIR150, and ZNF154 have been reported to exhibit differential expression between cirrhosis patients and HCC patients, suggesting their potential as biomarkers for early-stage HCC [16, 19, 20]. We conducted a differential methylation analysis on the methylation profiles of 430 HCC patients and 747 healthy individuals from TCGA database. As shown in Fig. 6C, we found significant discrepancy in methylation levels between these two groups at CpG sites within the genomic regions covered by the previously identified 13 significant DMRs. These 13 DMRs consistently exhibited hypermethylation in HCC patients compared to healthy population. In summary, this study confirms both the universality and accuracy of these selected 13 significant DMRs differences, suggesting their potential as biomarkers to discriminate early-stage HCC patients from healthy individuals. Supplementary materials contained additional analysis results comparing None cancer vs. HCC (Fig. S2) and None cancer vs. Early stage HCC (Fig. S3).

### Biomarkers for HBV-related HCC carcinogenesis

**Fig. 7:**
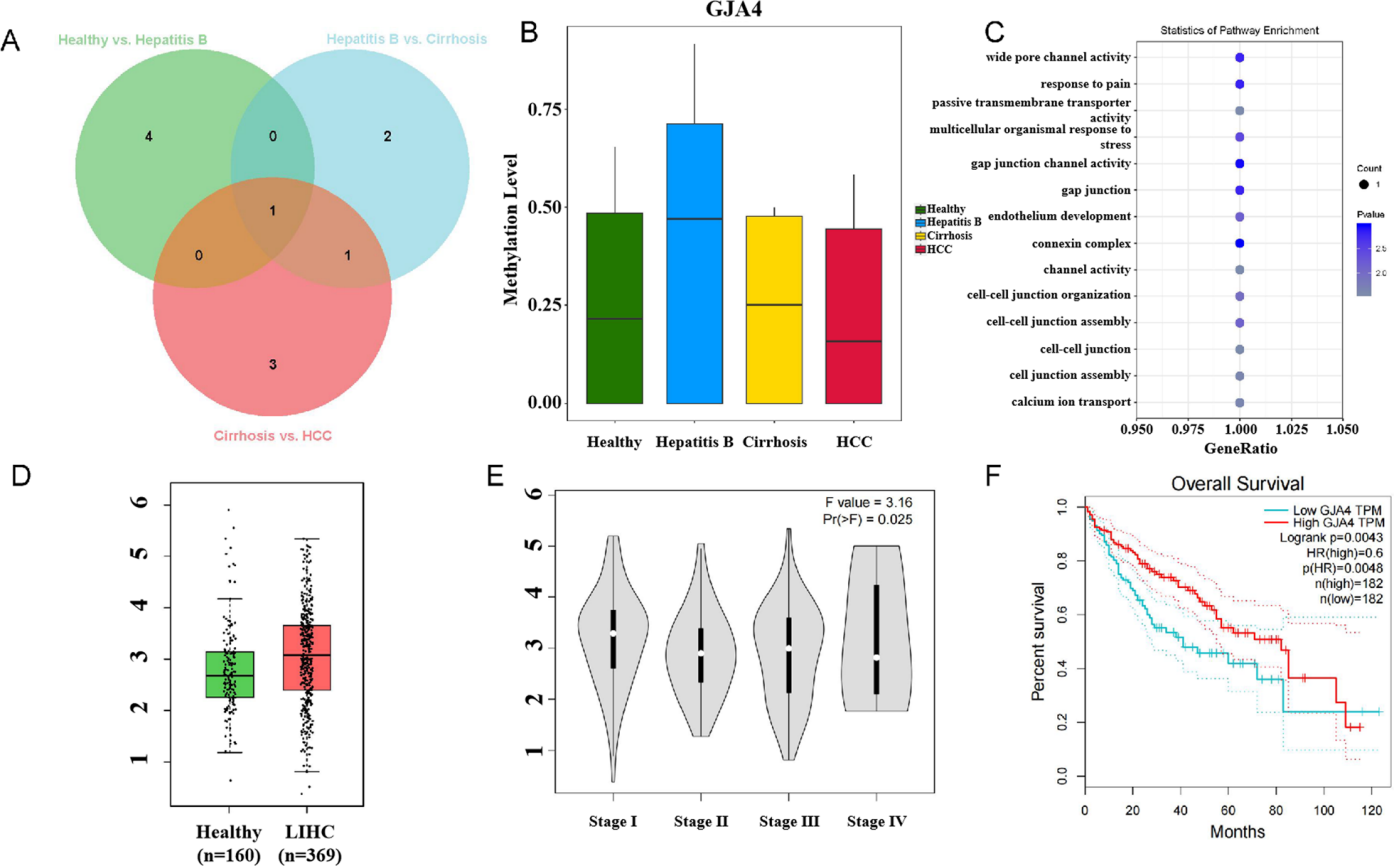
Discovery and validation of biomarkers for HBV-related HCC carcinogenesis. **A** The intersection of DMRs between three groups. **B** The methylation status of the GJA4 gene in the groups of healthy individuals, HBV patients, cirrhotic patients, and HCC patients. **C** The functional annotation bubble chart of GJA4. **D** The expression levels of the GJA4 gene in healthy individuals and HCC patients from TCGA database, with the vertical axis representing the gene expression value log2(TPM + 1). **E** The expression levels of the GJA4 gene in HCC patients at different stages, with the vertical axis representing the gene expression value log2(TPM + 1). **F** The correlation between GJA4 gene expression and patient survival rate

By conducting differential methylation analysis across three comparison groups, we identified a significant DMR that persists throughout the entire process of HBV-related HCC carcinogenesis (Fig. 7A). Gene annotation located this DMR to be within the promoter region of the GJA4 gene. In comparison to that of the healthy control group, methylation level of GJA4 were found to be upregulated in both Hepatitis B and cirrhosis groups while downregulated in HCC group, showing dynamic changes during the progression of HBV-related HCC carcinogenesis (Fig. 7B). The functional enrichment analysis of GJA4 revealed its involvement in oncogenic signaling pathways, including those associated with transcriptional regulation and cell fate decision (Fig. 7C). Previous studies have demonstrated the potential of GJA4 as a prognostic and therapeutic biomarker for HCC [21]. The expression profile of the GJA4 was investigated using TCGA gene expression data, revealing a significant upregulation of GJA4 expression in the HCC patient group compared to the healthy group (Fig. 7D). The gene expression profile of GJA4 aligned with the methylation profile of GJA4 (Fig. 7B), further supporting the selection of GJA4 as a biomarker for HBV pathology.

In terms of HCC staging, as the HCC stage increased, the expression of the GJA4 gene gradually decreased (Fig. 7E), indicating a certain regulatory role of GJA4 expression in HCC progression. And we also observed a significant correlation between lower GJA4 gene expression and decreased overall survival rates among HCC patients. This observation is consistent with the pattern of GJA4 gene expression in HCC staging, where lower GJA4 gene expression corresponds to advanced HCC stages and lower patient survival rates within a certain range. In conclusion, we propose that GJA4 may serve as a potential biomarker for HBV-related HCC carcinogenesis, as well as for the prognosis of HCC.

## Discussion

Early detection is the most effective way to improve the prognosis of HCC. Unfortunately, current diagnostic strategies for HCC, both serum AFP and ultrasound, lack sufficient sensitivity and specificity, especially for early HCC. A substantial body of research has demonstrated that cfDNA methylation markers hold significant clinical application potential for the early detection of HCC. Several promising screening models for HCC cfDNA methylation have been proposed. Guo et al. [6] used genome-wide methylation analysis to obtain 20 DMRs that specifically distinguished HCC tumors from surrounding tissues and healthy plasma, and incorporated them into multi-site qMSP assays to construct a HepaAiQ model. When detecting HCC in a cohort of 523 participants, the HepaAiQ model had an AUC of 0.940 and a sensitivity of 84.4%. When evaluated in an independent test set, the sensitivity of the HepaAiQ model was 70.8% in 65 BCLC stage 0/ a HCC patients. Zhao et al. [7] used plasma methylated GNB4 and Riplet as a novel double-labeled panel for the detection of HCC. In plasma, the sensitivity, specificity and AUC of this method for HCC at any stage were 84.39%, 91.92% and 92.51%. The sensitivity of the double-labeled panel to stage I HCC was 78.26%, higher than that of alpha-fetoprotein (AFP) 47.83%, and the sensitivity to a single tumor (size ≤ 3cm) was 70.27%. Current research has substantiated the potential of cfDNA methylation as a diagnostic marker for HCC; however, its sensitivity for the early detection of HCC remains to be enhanced.

In this study, four prediction models were constructed and then validated to discriminate between different groups of participants with considerable accuracy. Among these four models, the T-A-HCC and T-A-eHCC both achieved AUC of 0.988, while others yielded lower accuracy with AUC of 0.899 for T-nonCancer-HCC and AUC of 0.770 for T-nonCancer-eHCC. The two models to discriminate healthy individuals from HCC patients outperforms the two models to discriminate all non-cancer participants from HCC patients, because the non-cancer participants consist of healthy individuals as well as patients with hepatitis B and cirrhosis which represent a variety of pathological conditions. And some cirrhosis patients are already in transition to HCC, whose methylation profiles should exert a certain degree of similarity to those of HCC patients. Additionally, the model T-nonCancer-HCC outperforms the model T-nonCancer-eHCC, which is probably due to the fact that the early-stage HCC patients are still in the initial phase of tumorigenesis and their cancer-specific methylation alterations are not as prevalent as those of the HCC patients at later stages, which is supported by our finding that methylation alterations increase as the HCC stage advances. Additionally, machine learning, the basis algorithm for building up the prediction models, is highly dependent on the input sample size. Therefore, enlarged sampling in future studies is recommended to achieve higher accuracy, especially for the discrimination of all non-cancer participants from early-stage HCC patients, which can better imitate the early detection of HCC in real world.

As suggested by numerous studies [22, 23], we set three major criterion for selection of significant DMRs from Cohort 2 to be candidate biomarkers including 1) the prediction models based on these DMRs as feature markers can achieve considerable accuracy; 2) related genes of these DMRs participate in the signal pathways involved in tumoriogenesis; 3) differential methylation between HCC patients and non-cancer population can be extensively found in genomic regions related to these DMRs. Eventually, all these significant DMRs met the three criterion above and were selected as candidate biomarkers for HCC detection.

Besides detection, staging is another aspect of this study. The methylation profiles of the three staging categories, early, middle and late, were clearly different, with relatively more hypomethylation in later stages. This finding is supported by numerous studies [24, 25] showing the fundamental epigenomic feature of cancer genomes, genome-wide hypomethylation together with local hypermethylation. The overall methylation levels along different chromosomes decreased as the stage increased, further supporting this scenario.

Additionally, we found that compared to healthy individuals, the methylation level of GJA4 in HBV patients significantly increased. As the disease progressed to cirrhosis, the methylation level of GJA4 decreased significantly. And as the carcinogenic process goes further to reach HCC, the methylation level of GJA4 continue to drop to be even lower than that of healthy people. Therefore, we speculate that the methylation level of GJA4 could serve as a potential marker for detection of Hepatitis B and a clue to investigate the dynamic progression of HBV-related HCC carcinogenesis, the detailed mechanism of which needs to be clarified in future studies.

Despite the significance of our detection model, there are still some shortcomings. First, the number of patients included in the cohort was relatively small, especially in the validation cohort. Second, the function of GJA4 in HCC has not been verified. Third, this study mainly focused on HBV-associated HCC and did not study the application of this model in other HCC types or other cancer species. Fourth, given the impact of socioeconomic effects on the cost of the desired frequency of testing for the intended population (once every 6 months), the current version of the test is not cost-effective enough from a socioeconomic perspective and may not meet the needs of real-world clinical diagnosis. Fifth, the study did not address potential batch effects between cohorts. In future, we will further increase the number of samples and the types of cancer; the function of GJA4 in HCC was verified by in vivo and in vitro experiments.

In summary, this study provides a proof of principle study for HCC detection and staging using plasma cfDNA methylomes profiling, yielding potential biomarkers for detection and even early detection of HCC. Furthermore, the dynamic alteration of methylation of GJA4 promoter may server as a molecular clue for the study of HBV-related HCC carcinogenesis and prognosis of HBV-HCC.

## Supplementary Information

Additional file1 (XLSX 37 KB)

Additional file2 (XLS 66 KB)

Additional file3 (DOCX 12 KB)

Additional file4 (DOCX 12 KB)

Additional file5 (DOCX 14 KB)

Additional file6 (DOCX 12 KB)

Additional file7 (DOCX 12 KB)

Additional file8 (DOCX 11 KB)

Additional file9 (DOC 68598 KB)

## Data Availability

Data are provided within the manuscript or supplementary information files.
