# Supplementary material for: A liquid biopsy approach detects HCC and identifies GJA4 as a potential biomarker for HBV-HCC via plasma cfDNA methylome profiling
Source: Clin Epigenetics. 2025 Jun 11;17:98. doi: 10.1186/s13148-025-01909-w (PMC12160355; doi:10.1186/s13148-025-01909-w)
Supplement: Supplementary file 3 — Additional file3 (DOCX 12 KB) [file 13148_2025_1909_MOESM3_ESM.docx]

**Table S1. Characteristics of Patient distribution**

| Characteristic | Patient distribution of Cohort 1 dataset (N=300) | Patient distribution of Cohort 2 and Cohort 3 dataset (N=150) | Patient distribution of Cohort 4 dataset (N=74) |
| --- | --- | --- | --- |
| Age | 48.5(IQR=12 to 82, N=146) | 55.3(IQR=21 to 85, N=150) | 52.1(IQR=21 to 85, N=74) |
| Sex  Male | N=300  234 | N=150  115 | N=74  43 |
| Female  BCLC  0  A  B  C | 66  14  31  23  50 | 35  9  16  12  25 | 31  1  10  7  11 |
| D | 22 | 13 | 3 |
