# Supplementary material for: A liquid biopsy approach detects HCC and identifies GJA4 as a potential biomarker for HBV-HCC via plasma cfDNA methylome profiling
Source: Clin Epigenetics. 2025 Jun 11;17:98. doi: 10.1186/s13148-025-01909-w (PMC12160355; doi:10.1186/s13148-025-01909-w)
Supplement: Supplementary file 4 — Additional file4 (DOCX 12 KB) [file 13148_2025_1909_MOESM4_ESM.docx]

Table S2. Prediction models using TBS data in Cohorts 3

| Models | Training set | Test set | Prediction result |
| --- | --- | --- | --- |
| T-A-HCC | 15 healthy individuals.  47 HCC patients. | 6 healthy individuals.  20 HCC patients. | TP 19, TN 5,  FP 1, FN 1 |
| T-nonCancer-HCC | 47 non-cancer individuals ( 14 healthy individuals, 19 hepatitis B patients, 14 cirhosis patients).  47 HCC patients. | 20 non-cancer individuals ( 4  healthy individuals, 9 hepatitis B patients, 7 cirhosis patients).  20 HCC patients. | TP 19, TN 17,  FP 3, FN 1 |
| T-A-eHCC | 15 healthy individuals.  15 early-stage HCC patients ( 5 at stage 0, 10 at stage A) . | 6 healthy individuals.  6 early-stage HCC patients (2 at stage 0, 4 at stage A). | TP 5, TN 5,  FP 1, FN 1 |
| T-nonCancer-eHCC | 15 non-cancer individuals ( 2 healthy individuals, 5 hepatitis B patients, 8 cirhosis patients).  15 early-stage HCC patients ( 3 at stage 0, 12 at stage A) . | 6 non-cancer individuals (2 healthy individuals, 3 hepatitis B patients, 1 cirhosis patients). 6 early-stage HCC patients ( 4 at stage 0, 2 at stage A) . | TP 5, TN 5,  FP 1, FN 1 |
