# Supplementary material for: A liquid biopsy approach detects HCC and identifies GJA4 as a potential biomarker for HBV-HCC via plasma cfDNA methylome profiling
Source: Clin Epigenetics. 2025 Jun 11;17:98. doi: 10.1186/s13148-025-01909-w (PMC12160355; doi:10.1186/s13148-025-01909-w)
Supplement: Supplementary file 5 — Additional file5 (DOCX 14 KB) [file 13148_2025_1909_MOESM5_ESM.docx]

Table S3. Significant DMRs of Healthy vs. HCC comparison from TBS data

| **Significant DMR** | **Type** | **Gene** |
| --- | --- | --- |
| chr1_6454731_6454831 | intronic | ESPN |
| chr1_6454842_6454942 | intronic | ESPN |
| chr1_6455256_6455436 | intronic | ESPN |
| chr1_47439275_47439662 | upstream | LINC01389(dist=1958) |
| chr1_90717867_90717967 | upstream | BARHL2(dist=565) |
| chr1_108660902_108661098 | UTR5 | HENMT1(NM_001102592:c.1209_1079delins0) |
| chr1_203629327_203629563 | intronic | ATP2B4 |
| chr1_235650660_235650777 | upstream | GNG4(dist=52) |
| chr10_100713833_100713977 | intergenic | HIF1AN(dist=153838),PAX2(dist=21419) |
| chr10_132785321_132785517 | downstream | INPP5A(dist=1841) |
| chr11_13009356_13009456 | UTR5 | RASSF10(NM_001080521:c.-221_-121delins0) |
| chr11_62709491_62709597 | intronic | HNRNPUL2-BSCL2 |
| chr12_51820971_51821235 | exonic | FIGNL2 |
| chr12_95548214_95548321 | UTR5 | USP44(NM_001278393:c.13982_13958delins0) |
| chr12_132904812_132905015 | intergenic | LOC101928530(dist=16241),ZNF605(dist=13291) |
| chr14_103189099_103189204 | upstream | LINC00605(dist=71) |
| chr14_102928437_102928636 | exonic | AMN |
| chr14_103273580_103273713 | intergenic | LINC00605(dist=84552),LOC105378183(dist=43571) |
| chr15_90956648_90956767 | exonic | RCCD1 |
| chr17_30971149_30971299 | exonic | RNF135 |
| chr17_38509977_38510103 | exonic | ARHGAP23 |
| chr17_72116532_72116632 | upstream | SOX9-AS1(dist=516) |
| chr17_77372725_77372825 | intronic | SEPTIN9 |
| chr17_77374096_77374299 | intronic | SEPTIN9 |
| chr17_61405228_61405424 | exonic | TBX2 |
| chr17_77372936_77373255 | intronic | SEPTIN9 |
| chr17_82986153_82986261 | intronic | B3GNTL1 |
| chr17_82986272_82986373 | intronic | B3GNTL1 |
| chr17_77373393_77373673 | exonic | SEPTIN9 |
| chr19_12195343_12195515 | intronic | LOC100289333 |
| chr19_57709013_57709113 | UTR5 | ZNF154(NM_001085384:c.-42_-142delins0) |
