# Supplementary material for: A liquid biopsy approach detects HCC and identifies GJA4 as a potential biomarker for HBV-HCC via plasma cfDNA methylome profiling
Source: Clin Epigenetics. 2025 Jun 11;17:98. doi: 10.1186/s13148-025-01909-w (PMC12160355; doi:10.1186/s13148-025-01909-w)
Supplement: Supplementary file 6 — Additional file6 (DOCX 12 KB) [file 13148_2025_1909_MOESM6_ESM.docx]

Table S4. Significant DMRs of Healthy vs. Early-stage HCC comparison from TBS data

| **Significant DMR** | **Type** | **Gene** |
| --- | --- | --- |
| chr1_6454842_6454942 | intronic | ESPN |
| chr1_90717867_90717967 | upstream | BARHL2(dist=565) |
| chr1_243482948_243483379 | intronic | SDCCAG8 |
| chr10_132785321_132785517 | downstream | INPP5A(dist=1841) |
| chr11_64713220_64713329 | exonic | NRXN2 |
| chr12_104303178_104303838 | UTR5 | EID3(NM_001008394:c.-196_-97delins0) |
| chr13_87672572_87672712 | upstream | MIR4500HG(dist=1609) |
| chr15_89496486_89496634 | exonic | RHCG |
| chr16_58463355_58463480 | upstream | NDRG4(dist=235) |
| chr17_3535641_3535741 | exonic | TRPV3 |
| chr19_49501252_49501361 | upstream | MIR150(dist=384) |
| chr19_53980080_53980184 | intronic | CACNG8 |
| chr19_57709013_57709113 | UTR5 | ZNF154(NM_001085384:c.-42_-142delins0) |
