# Supplementary material for: A liquid biopsy approach detects HCC and identifies GJA4 as a potential biomarker for HBV-HCC via plasma cfDNA methylome profiling
Source: Clin Epigenetics. 2025 Jun 11;17:98. doi: 10.1186/s13148-025-01909-w (PMC12160355; doi:10.1186/s13148-025-01909-w)
Supplement: Supplementary file 7 — Additional file7 (DOCX 12 KB) [file 13148_2025_1909_MOESM7_ESM.docx]

Table S5. Significant DMRs of Non cancer vs. HCC comparison from TBS data

| **Significant DMR** | **Type** | **Gene** |
| --- | --- | --- |
| chr10_132785321_132785517 | downstream | INPP5A(dist=1841) |
| chr12_95548878_95548978 | intronic | USP44 |
| chr12_132904812_132905015 | intergenic | LOC101928530(dist=16241),ZNF605(dist=13291) |
| chr14_102928437_102928636 | exonic | AMN |
| chr14_103273580_103273713 | intergenic | LINC00605(dist=84552),LOC105378183(dist=43571) |
| chr15_90956648_90956767 | exonic | RCCD1 |
| chr17_30971149_30971299 | exonic | RNF135 |
| chr17_77373393_77373673 | exonic | SEPTIN9 |
| chr10_132785321_132785517 | downstream | INPP5A(dist=1841) |
