# Supplementary material for: A liquid biopsy approach detects HCC and identifies GJA4 as a potential biomarker for HBV-HCC via plasma cfDNA methylome profiling
Source: Clin Epigenetics. 2025 Jun 11;17:98. doi: 10.1186/s13148-025-01909-w (PMC12160355; doi:10.1186/s13148-025-01909-w)
Supplement: Supplementary file 8 — Additional file8 (DOCX 11 KB) [file 13148_2025_1909_MOESM8_ESM.docx]

Table S6. Significant DMRs of Non cancer vs. Early-stage HCC comparison from TBS data

| **Significant DMR** | **Type** | **Gene** |
| --- | --- | --- |
| chr1_20343390_20343490 | exonic | VWA5B1 |
| chr12_95548878_95548978 | intronic | USP44 |
