# Supplementary material for: A liquid biopsy approach detects HCC and identifies GJA4 as a potential biomarker for HBV-HCC via plasma cfDNA methylome profiling
Source: Clin Epigenetics. 2025 Jun 11;17:98. doi: 10.1186/s13148-025-01909-w (PMC12160355; doi:10.1186/s13148-025-01909-w)
Supplement: Supplementary file 9 — Additional file9 (DOC 68598 KB) [file 13148_2025_1909_MOESM9_ESM.doc]

**Supplementary material**

**1. Prediction models for HCC detection based on TBS data**

In Chort 2, a total of 8 significant DMRs were identified in the comparison between None cancer and HCC, all exhibiting hypermethylation (Fig. S1A). Additionally, 2 significant DMRs showing hypermethylation were found in the comparison between None cancer and Early-stage HCC (Fig. S1E). In Cohort 3, the T-nonCancer-HCC model achieved a sensitivity of 85.00%, a specificity of 85.71%, and an AUC of 0.948 (Fig. S1B). The prediction scores was depicted in Fig. S1C. The T-nonCancer-eHCC model achieved a sensitivity and specificity of 83.33% each, with an AUC value of 0.889 (Fig. S1F). The prediction scores was shown in Fig. S1G. Subsequently, utilizing the same significant DMRs as feature markers, we validated the T-nonCancer-HCC model by employing all non-cancer individuals and HCC patients from Cohort 3 as the training set, along with all non-cancer individuals and HCC patients from Cohort 4 as the test set. Remarkably, this validation yielded an impressive AUC of 0.899 (Fig. S1D). The model T-nonCancer-eHCC was validated using all non-cancer individuals and early-stage HCC patients in Cohort 3 as the training set, and all non-cancer individuals and early-stage HCC patients in Cohort 4 as the test set, achieving an AUC of 0.770 (Fig. S1H).

**Figure S1. Development of the T-nonCancer-HCC and** **T-nonCancer-eHCC prediction models using TBS data.** (A) Decomposition analysis of hyper- or hypo-methylation patterns in significant DMRs between None cancer and HCC groups; (B) Prediction scores for the T-nonCancer-HCC model; (C) ROC curve for building up of the T-nonCancer-HCC model; (D) ROC curve for validation of the T-nonCancer-HCC model; (E) Decomposition analysis of hyper- or hypo-methylation patterns in significant DMRs between None cancer and Early-stage HCC groups; (F) Prediction scores for the T-nonCancer-eHCC model; (G) ROC curve for building up of the T-nonCancer-eHCC model; (H) ROC curve for validation of the T-nonCancer-eHCC model.

**2. HCC specific methylation markers were identified based on significant DMRs**

Based on the comparison between Non cancer and HCC, we identified 8 significant DMRs. Subsequently, using Annovar software, we annotated 10 genes associated with these DMRs (Table S5). Gene Ontology (GO) enrichment analysis revealed their involvement in multiple carcinogenic signaling pathways (Fig. S2A-S2B). Furthermore, differential methylation analysis of methylation profiles from 430 HCC patients and 747 healthy individuals obtained from TCGA demonstrated significant differences in methylation levels at two CpG sites within the genomic region covered by the identified DMRs (Fig. S2C-S2D). These findings supported the potential candidacy of these significant DMRs as biomarkers for distinguishing HCC patients from Non-cancer population.


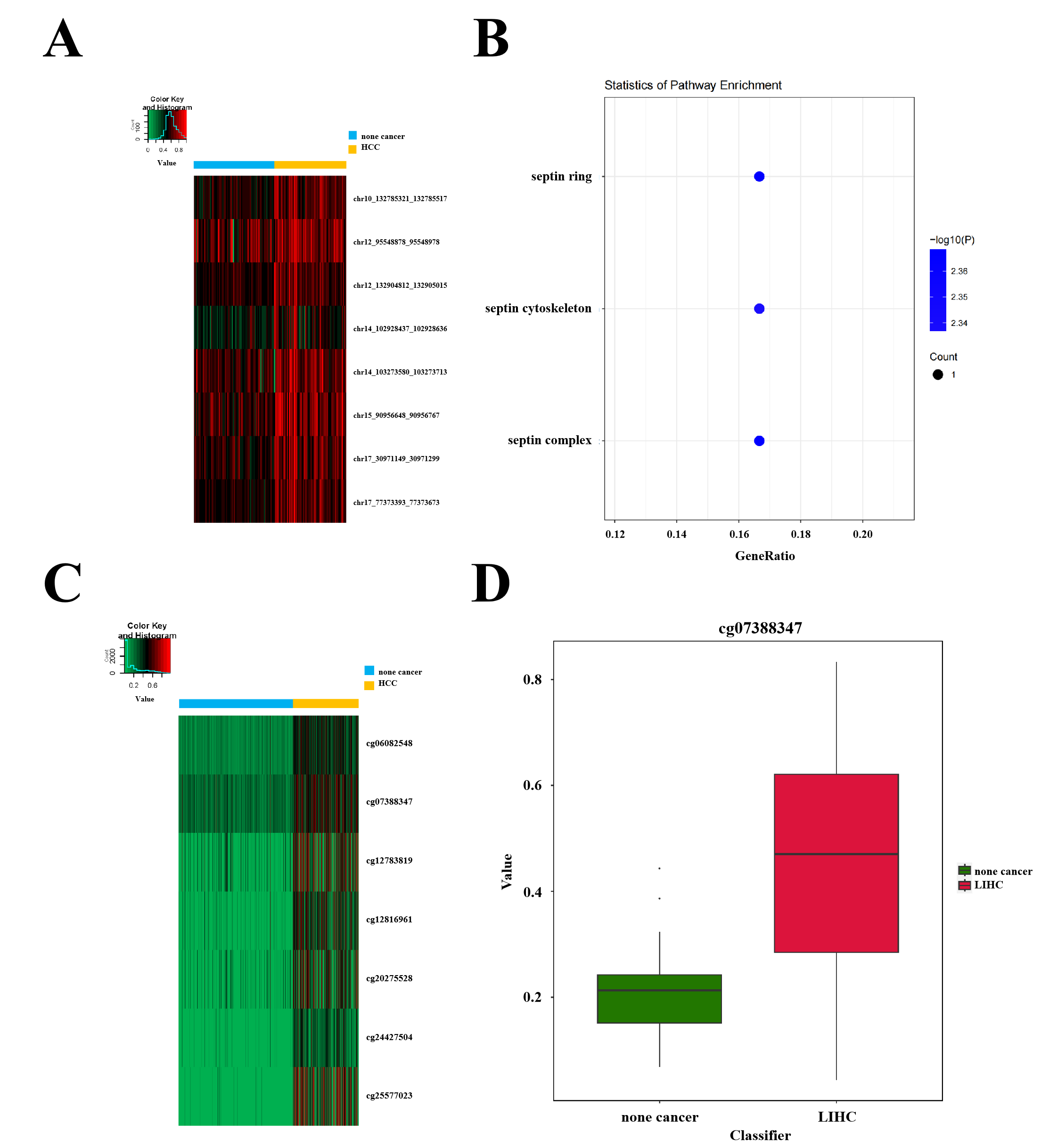


**Figure S2. Biological interpretation of candidate biomarkers for distinguishing HCC patients from Non cancer individuals**. (A) Boxplot illustrating the unsupervised clustering of differential methylation levels in the 8 candidate biomarkers (significant DMRs) between a cohort of 70 non-cancer participants and 67 HCC patients encompassing all stages, as identified from TBS data; (B) Enrichment analysis of gene ontology for candidate biomarker; (C) Heatmap illustrating the unsupervised clustering of differential methylation status at CpG sites within the regions of significant DMRs identified above, comparing 747 healthy individuals with 430 HCC patients; (D) Boxplot for the methylation values of 747 healthy people and 430 HCC patients at CpG site cg07388347.

Based on the identification of two significant differentially methylated regions (DMRs) between Non cancer and early-stage HCC, two genes were annotated using Annovar software (Table S6). Subsequent Gene Ontology (GO) enrichment analysis revealed their involvement in carcinogenic signaling pathways, specifically those related to transcription regulation, cell fate determination, and the regulation of oncogenes and tumor suppressor genes (Fig. S3A-S3B).


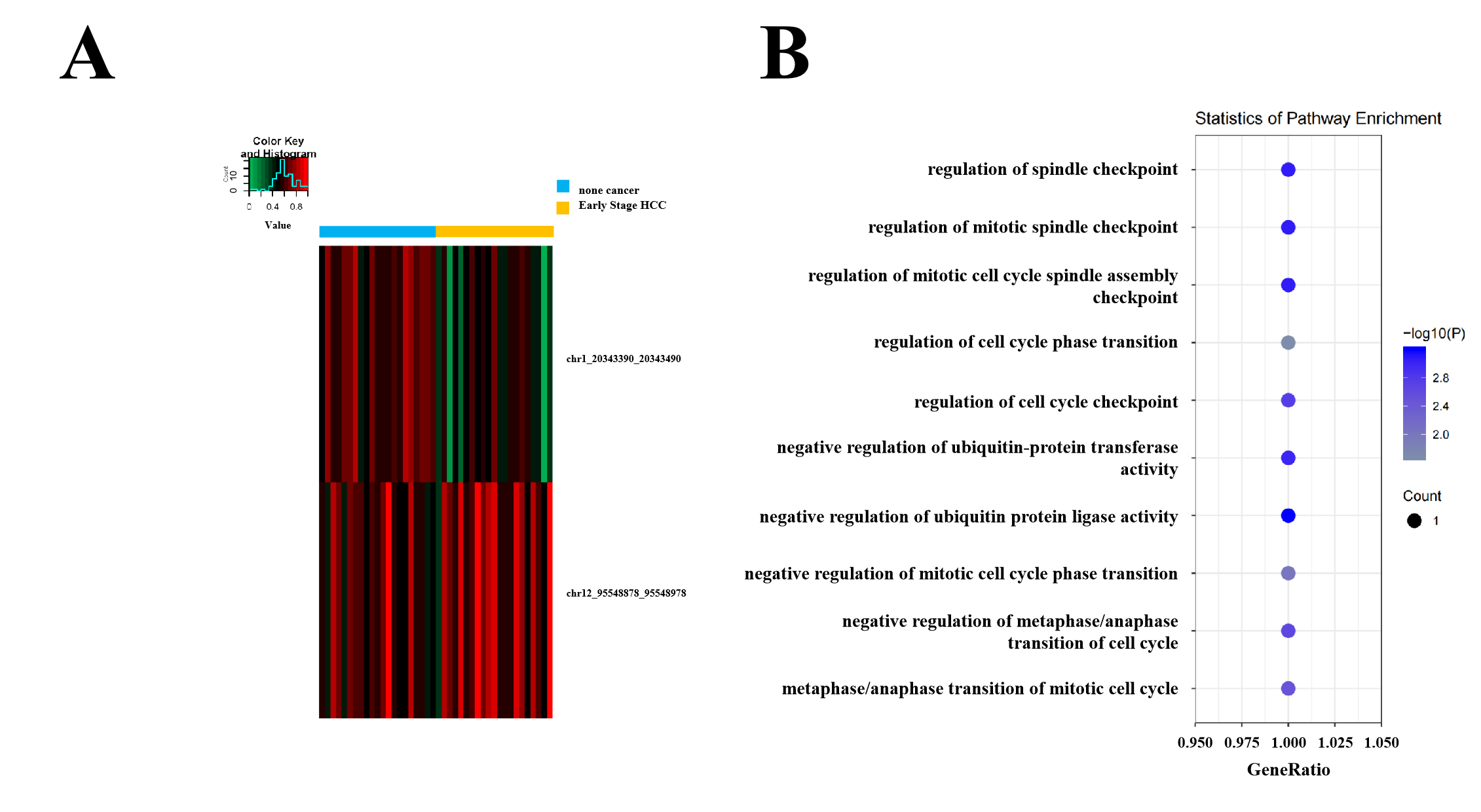


**Figure S3. Biological interpretation of candidate biomarkers for discriminating early-stage HCC patients from Non cancer population.** (A) Boxplot illustrating unsupervised clustering of differential methylation levels in the 2 identified candidate biomarkers (significant DMRs) between 70 Non cancer participants and 21 early-stage HCC patients, as determined from TBS data; (B) Enrichment analysis of gene ontology for candidate biomarker.
